# Neural Correlates of Mental Rotation in Preschoolers With High or Low Working Memory Capacity: An fNIRS Study

**DOI:** 10.3389/fpsyg.2020.568382

**Published:** 2020-12-10

**Authors:** Jinfeng Yang, Dandan Wu, Jiutong Luo, Sha Xie, Chunqi Chang, Hui Li

**Affiliations:** ^1^Shenzhen University, Shenzhen, China; ^2^Macquarie University, Sydney, NSW, Australia; ^3^The University of Hong Kong, Pokfulam, Hong Kong

**Keywords:** neural network, mental rotation, working memory, Chinese preschoolers, functional near-infrared spectroscopy

## Abstract

This study explored the differentiated neural correlates of mental rotation (MR) in preschoolers with high and low working memory capacity using functional near-infrared spectroscopy (fNIRS). Altogether 38 Chinese preschoolers (*M* = 5.0 years, *SD* = 0.69 years) completed the *Working Memory Capacity* (WMC) test, the *Mental Rotation* (MR), and its Control tasks (without MR). They were divided into High-WMC (*N_1_* = 9) and Low-WMC (*N*_2_ = 18) groups based on the WMC scores. The behavioral and fNIRS results indicated that: (1) there were no significant differences in MR task performance between the High-WMC (*M*_*mr*_ = 23.44, *SD* = 0.88) and Low-WMC group (*M_*mr*_* = 23.67, *SD* = 0.59); (2) the Low-WMC group activated BA6, BA8, BA 9, and BA 44, whereas the High-WMC group activated BA8, BA10 and BA 44 during mental rotation; (3) significant differences were found in the activation of BA44 and BA9 between the High-WMC and Low-WMC groups during mental rotation; and (4) the High-WMC and Low-WMC groups differed significantly in the activation of BA 9 and BA10 during the control tasks, indicating that both areas might be responsible for the group differences in working memory.

## Indroduction

Mental rotation (MR) has been extensively employed to evaluate early cognitive development (e.g., Hoppe et al., 2012), as it is a cognitive process in which participants have to form a mental image of the target assemblage and align it with the other assemblage by rotating this image (Shepard and Cooper, 1982, for a review; Zacks, 2008, for a meta-analytic review). This cognitive process is based on the processing of visual or object working memory (Hyun and Luck, 2007), thus has substantially involved the frontal cortex (BA 9, BA10), premotor cortex (BA 6), parietal cortex (BA 40, BA 44) (Cohen et al., 1996; Jordan et al., 2001; Schöning et al., 2007). All these studies, however, were conducted with adult participants. Recently, Wu et al. (2020) examined the neural correlates of MR in preschoolers using functional near-infrared spectroscopy (fNIRS) and found that BA6, BA9, BA44 were involved in the MR processing. But the role of working memory in preschoolers’ MR has not been explored, even though it is substantially engaged in mental rotation (Gauthier et al., 2002; Hyun and Luck, 2007). Therefore, this study will fill the gap by duplicating and extending the MR tasks by Wu et al. (2020) to explore the relationship between working memory and mental rotation.

### The Neural Correlates of Mental Rotation

Using fMRI, Cohen et al. (1996) found that the frontal cortex (BA 9, BA 44, BA 46), premotor cortex (BA 6), and parietal cortex (BA 7, BA 40) were significantly activated during mental rotation, and some adult cases showed noticeable activation in BA 39 and BA 19. Later, Richter et al. (2000) conducted an fMRI study with Shepard and Metzler’s classic task and found a bilateral involvement of the superior parietal lobule, lateral premotor area, and supplementary motor area in the very act of mental rotation. They also found activation in the left primary motor cortex, which seemed to be associated with the right-hand button press at the end of the task period. This was verified by Windischberger et al. (2003), who found that the button pressing caused activation in the primary motor cortex (BA 4) and supplementary motor area (SMA, BA 6) while the parietal cortex (BAs 5, 7, 39, 40) and mesial regions rostral to the supplementary motor area were recruited for the actual mental rotation process.

Harris and Miniussi (2003) employed repetitive transcranial magnetic stimulation (rTMS) and found that the right superior posterior parietal lobe might play an essential role in mental rotation. However, this study could not rule out the role of the left posterior parietal lobe in mental rotation. Accordingly, Kucian et al. (2007) investigated the maturing neural network for mental rotation by comparing brain activation in 20 children and 20 adults using fMRI. They found that adults exhibited more robust activation in the left intraparietal sulcus compared to children. This finding suggests a shift of activation from a predominantly right parietal activation in children to a bilateral activation pattern in adults.

Later, Milivojevic et al. (2008) studied the brain regions involved in mental rotation by assessing the fMRI activation during a letter-digit judgment task. They found that the mental rotation was sub-served by a bilateral frontoparietal network. Therefore, they suggested that the hemispheric asymmetries found in the parity-judgment tasks might reflect visuospatial processing other than mental rotation itself, which could be sub-served by a bilateral frontoparietal network. Later, Zhang et al. (2009) investigated the interactive cortical networks involved in Chinese Character MR tasks using the Partial directed coherence (PDC) analysis. They found that during MR of Chinese character (1) cortical interactive networks changed according to task difficulty, and (2) the right hemisphere played an initiating role in bilateral cortical activation. However, all these neuroimaging studies have not explored the role of working memory in mental rotation, especially in the preschoolers who are gradually acquiring the cognitive ability of mental rotation. Therefore, this study will address this research gap with near-infrared spectroscopy technology.

### Working Memory and Mental Rotation in Preschoolers

Neuroimaging studies have consistently found that mental rotation would involve spatial or object working memory (Gauthier et al., 2002; Hyun and Luck, 2007). Initially, Carpenter et al. (1999) found an extensive activation of both dorsal and ventral stream areas during an MR task compared to a control task. However, only the dorsal stream activation was strongly dependent on the degree of rotation. Later, Hyun and Luck (2007) found that prefrontal areas (BA 9 and BA 10) appeared to be involved in both spatial and object working memory and especially to be responsible for the control and manipulation of information in working memory, rather than being the “storeroom” of the spatial and object information. As mental rotation requires both the storage and manipulation of spatial and object information, precisely the neural function of prefrontal areas (BA 9 and BA 10), we tend to believe that BA 9 and BA 10 might play a critical role in the processing of mental rotation tasks. Therefore, this study will explore the role of BA 9 and BA10 in mental rotation processing in a group of Chinese preschoolers.

Preschoolers’ mental rotation, however, has been rarely explored by neuroimaging studies. Most of the existing studies simply adopted the traditional behavioral paradigms. For instance, Frick et al. (2013) assessed individual differences in children’s mental rotation abilities between 3.5 and 5.5 years of age. They found that: (1) children’s error rates and response times increased linearly with increasing angular disparity by the age of 5 years; (2) 4-year-olds were found to respond at a chance for all angular disparities; (3) both manual and observational experience increased the response accuracy of 5-year-olds, but there was no effect on 4-year-olds. These results indicated that the mental rotation paradigm’s successful application should be restricted to children 5 years or older. To challenge this age limitation, Krüger et al. (2014) developed a new research paradigm allowing for the measurement and interpretation of reaction time with 3- to 6-year-olds. They presented a stimulus configuration on a touchscreen and asked preschoolers to bring a rotated stimulus into an upright position using the shortest path. They found that the 3- and 4-year-olds performed reliably above the chance level, but only 5- and 6-year-olds could correctly complete the tasks.

Naseer and Hong (2015) conducted a systematic review and found that fNIRS showed a significant advantage in studying the prefrontal cortex due to no hair in detecting the cognitive tasks like mental arithmetic, music imagery, so on. In extracting features related to the desired brain signal, the mean, variance, peak value, slope, skewness, and kurtosis of the noised-removed hemodynamic response were used. Therefore, they believed that fNIRS would be more widely used to monitor the occurrence of neuro-plasticity after neuro-rehabilitation and neuro-stimulation. Recently, Wu et al. (2020) examined MR’s neural correlates in preschoolers using fNIRS and the mental rotation paradigms developed by Krüger et al. (2014). They found that the 48 Chinese preschoolers (*M* = 66.15 months) could complete the behavioral tasks and be classified into Low and High MR performance groups. And the fNIRS results indicated that BA 44 might be one of MR’s core neural correlates in preschoolers, and BA 6 and BA 9 might also be involved in MR processing under a compensatory mechanism. However, the major finding that BA44 was the “neural correlate” (core brain area) of mental rotation might be confounded by other factors, such as it is also in charge of hand movements (Gallagher et al., 2002). Future studies should control hand movements using a control task to reaffirm the precise contribution of BA44 in MR. Also, limited by the research design, Wu et al. (2020) study did not explore working memory’s role, leaving a research gap to be filled by this study. Therefore, we have endeavored to address the following questions in this study:

1. Are there any relationships between the preschoolers’ performance in the working memory and mental rotation tasks?
2. Are there any significant differences in the neural correlates of mental rotation between the preschoolers with high and low working memory capacity?
3. What are the brain areas involved in the mental rotation according to the fNIRS evidence?

## Materials and Methods

### Sample

Altogether 42 right-handed preschoolers participated in this study before the outbreak of COVID-19 in late January 2020. Parents of these children were informed verbally of the purpose of the research and the fNIRS experiments’ safety before they signed the written consent form. The University Ethics Committee approved the experiment and ethical clearance. Among the 42 children, four failed to complete the tests and were thus excluded, resulting in a final sample of 38 children (aged between 4 and 6.3 years, *M* = 5.0 years, *SD* = 0.686 years).

### Instrument

#### Working Memory Capacity (WMC) Test

The Missing Scan Task (MST) (Roman et al., 2014) was adopted in this study. MST has been validated by Roman et al. (2014) as a workable and reliable measure of working memory in preschool children (3–6 years in age). Recently, Jusienė et al. (2020) have adopted MST to test 190 preschool children’s working memory and further verified it with sound psychometric properties. Among 65 Beanie Babies (small animal-shaped bean-filled bags), 15 were chosen and used as test stimuli in this study. Examples of animals in the test set included turtle, pig, cow, and duck. Each Beanie Baby was named by the participant (i.e., turtle, pig, cow) to prevent the need to learn new vocabulary; therefore, the participating child could consistently label this animal and did not refer to another animal the same set by the same name. To assess their existing knowledge of the animal names in the stimulus set, we asked the participants to name pictures of each Beanie Baby animal before carrying out the MST. If the participant did not recognize the animal, it would not be included in the test set.

#### Mental Rotation (MR) Tasks

**FIGURE 1:**
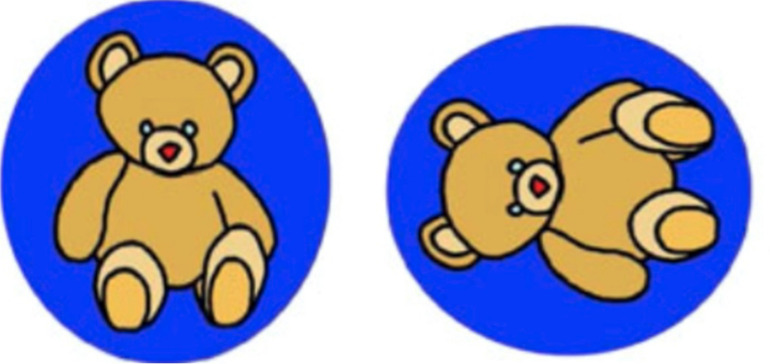
The mental rotation (MR) task.

The MR task in this study was modified from the version developed by Krüger et al. (2014), containing 24 pictures. As shown in Figure 1, the test and target stimuli were physical pictures printed on paper cards and test books. One stimulus was not rotated (target stimulus, left side) while the other one was rotated clockwise to one of the following angles: 45°, 90°, 135°, 225°, 270°, or 315° (test stimulus; right side). As many of the participants had no experience using a PC desktop, no screen task was employed in this study. In the training and testing sessions, the paper card with a picture of a duck was presented to the participants to show how to rotate it and make it stand up (using the shortest path). All the test and target materials were presented to the participants on a table.

#### Control Tasks

**FIGURE 2:**
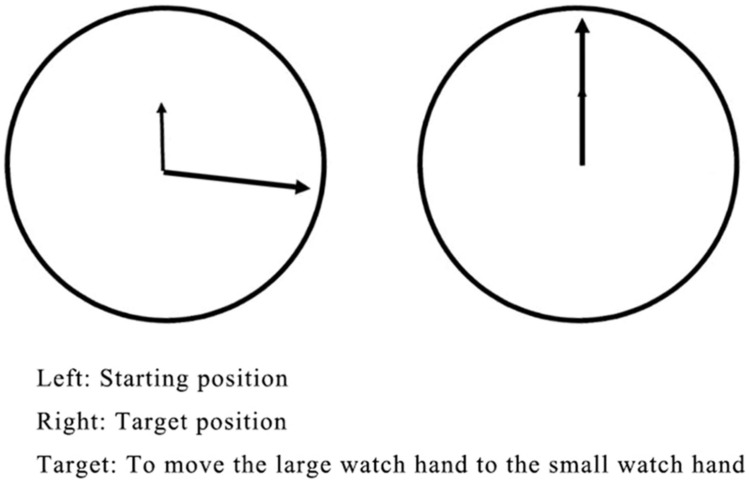
The control task.

To control for the effects of hand movement and movement planning, a set of tasks was conducted to ask the participants to perform a manual rotation movement similar to that in the MR task but without a decision about the movement’s direction. The stimulus material consisted of a clock-like schematic drawing. As shown in Figure 2, the small hand of the “clock” was always set to 12 o’clock (target position). The big hand was set between 12 and 6 o’clock (starting position). The angle between the two watch hands varied in 14 different degrees (80° and 160° for training; 15°, 30°, 45°, 60°, 75°, 90°, 105°, 120°, 135°, 150°, 165°, and 180° for testing). The big hand could be moved toward the small watch hand (target position) counter-clockwise only.

### Procedure

#### Cap Placement

First, the participants were informed about the fNIRS experiment in terms of an invitation to play games. The participants were encouraged to report any uncomfortable feelings so that the technician could adjust the cap for them. The participants were allowed to quit anytime during the experiment. Once the participants gave their consent to participate, the experimenter read a picture book with them while an experienced technician assisted them in putting on the fNIRS cap.

Second, the technician performed the cap placement, hair manipulation, and tossing and the installation of optodes (based on the 10/20 system). The cap placement procedure involved making general head measurements to decide the cap’s size to be used for each participant. Both small (S) and extra-small (XS) fNIRS caps accompanied by the fNIRS instrument (Oxymon Mk III, Artinis, Netherlands) were used for the Chinese preschoolers in this study. The cap is a highly stretchable soft headwear covering the entire head, with prefixed locations for optodes, much like an EEG cap. It has digitized optode positions to illustrate the brain areas being studied. Additional colorful hairbands were used to keep the cap in place and to prevent slipping. As the cap placement procedure took approximately half an hour, children were engaged in storybook reading with an experienced preschool teacher during this period.

#### Working Memory Assessments

The participant with the fNIRS cap sat across from the experimenter, where a small cardboard house was placed on the table facing the participant. Out of the participant’s line of sight, a back-pack was placed under the table, which contained the 15 animal-shaped Beanie Babies. The experimenter explained to the participant that they were going to play a memory game. The experimenter brought out two randomly selected Beanie Babies and placed them on the table in front of the participant. The two animals represented a memory set size of two and were used as the training and practice set for each child. The participant was asked to name and remember the two animals, as “they would go inside the house where the participant would not be able to see them anymore,” and when they came back out of the house, one of the animals would be missing. Each child was given approximately 10 s to look at the animals in the memory set and name them before the experimenter placed them inside the house. Two or three seconds later, one Beanie Baby was brought back (chosen at random), and the participant was asked, “which one is missing?” The participant had to display an understanding of the instructions before proceeding with the MST. If the participant were unable to demonstrate an understanding, he/she would not continue with the MST. All the children reported in this study have completed the practice set and proceeded to the test sets.

The memory set size began with three animals and increased in length by one animal each time when the participant correctly reported the missing item. After one correct trial at a given set size was completed, the memory set size was increased by one item. If the participant incorrectly named the missing animals, the same memory set size was tested again with a new test item. In both training and test trials, the participants were shown the missing animal after each trial regardless of the answer’s correctness. The MST concluded when the participant either failed to correctly name the missing animal on two trials of the same memory set size or successfully completed a set size of 10. The animals in each memory set were always novel and were randomized for each set size without replacement. The presentation order was also randomized for each child. Working memory capacity (WMC) was defined as the most extended set size that the participant could correctly scan with no errors.

#### MR Task

The training session of the MR task consisted of four trials. The experimenter explained to children how the first trial should be performed with the following instructions: “*Here is an upright bear [experimenter pointing the target stimulus on the left] and here is a duck falling on its side [pointing the duck on the right]. Now let us help this bear get back on its feet as soon as possible. We can help him get up this way [rotating the test stimulus via the shortest route]. However, if you do it like this [rotating the test stimulus via a more distant route], the bear will be unhappy. So, please do not do it this way.*” The participants were then asked to perform the remaining three training trials, during which the experimenter corrected them upon any mistakes. When a child made a mistake, i.e., chose the more distant route, the experimenter would repeat the original instructions and ask the participant to repeat the corresponding trial. If the participant made the same mistake again, he/she would be asked to perform all training trials again.

**FIGURE 3:**
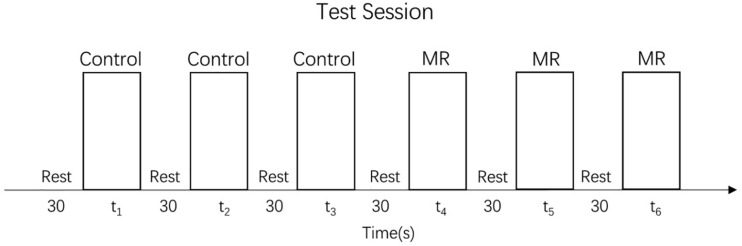
Test session.

The test session of the MR task consisted of 24 trials (24 different stimulus pairs) divided into three task blocks (8 trial/block), each of which preceded a rest block (see Figure 3). In each trial, a target (unrotated) stimulus was presented on the left side of the table while a test stimulus, rotated to one of the six angles mentioned above, was presented on the right side of the table. The trials were randomized in each block. No trials were repeated. As in the training session, the participants were instructed to rotate the test stimulus to match the target stimulus. No help or further instructions were given.

#### Control Task

The control task’s training consisted of four trials with the large hand starting randomly at either 80° or 160°. At the start of the training phase, the experimenter showed children the stimulus material and explained that it would be the participant’s task to move the large watch hand to the small watch hand. Then the experimenter solved the first trial for the participant by “dragging” the large hand counter-clockwise to the small hand. Afterward, children were asked to do so by themselves, but the experimenter offered assistance and answered their questions. If children made a mistake, the instruction was repeated.

The control task test consisted of 36 trials divided into three blocks, each of which preceded a rest block (see Figure 3). The set of stimuli was the same in every block and consisted of 12 different angles (15°, 30°, 45°, 60°, 75°, 90°, 105°, 120°, 135°, 150°, 165°, and 180°) presented in random order. Precisely as in the training session, children were always asked to move the big hand to the small hand, but no help or further instructions were given.

### Data Acquisition, Processing, and Analysis

In this study, a multiple-channel fNIRS system (OxyMon Mk III, Artinis, Netherlands) was used to simultaneously measure the concentration changes of oxygenated hemoglobin (HbO), deoxygenated hemoglobin (HbR), and total hemoglobin (HbT) in the participants. Two wavelengths in the near-infrared range, namely 760 and 850 nm, were used to measure the changes in optical density, which were then converted into changes in the concentration of HbO and HbR using the modified Beer-Lambert law.

**FIGURE 4:**
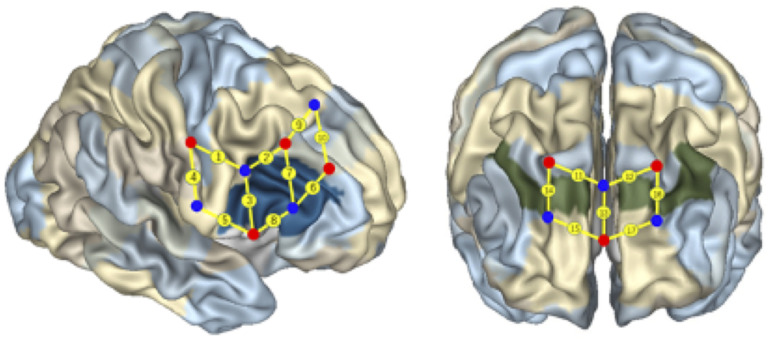
Localization of regions of interest. The numbers on small spheres on the brain map indicate the 17 channels. The channel localization was based on the upper central probe, which was anchored at Fz according to the international 10–20 system and was located at the midpoint between channels numbers 11 and 12. The channels 1 and 9 were located in BA 6, the channel 10 was located in BA 8, the channels 11, 12, 14, 16 were located in BA 9, and the channels 13, 15, 17 were located in BA 10, the channel 4 was located in BA 40, the channels 2, 3, 5, 6, 7, and 8 were located in the right IFC (BA 44).

Seventeen fNIRS channels were used and located following the international 10/20 system for EEG, with a 2.5 cm distance between adjacent emitter-detector pairs. The regions of interest (ROIs) were located at Brodmann Areas (BAs) 6, 8, 9, 10, 40, and 44 (see Figure 4). Previous studies have shown that these areas might be activated during cognitive shifting, mental rotation, and other preschoolers’ cognitive activities (Moriguchi and Hiraki, 2009; Wu et al., 2020). Ten channels were located in the right frontal cortex, and seven channels were located in the prefrontal cortex (see Figure 4). In particular, the channels 1 and 9 were located at BA 6, channel 10 at BA 8, the channels 11, 12, 14, 16 at BA 9, and channels 13, 15, 17 at BA 10, channel 4 at BA 40, and channels 2, 3, 5, 6, 7, 8 were located at BA 44 in the right inferior frontal cortex.

A subject-specific differential pathlength factor (DPF) constant was calculated based on the age of each subject (Duncan et al., 1996). And the sampling rate was set at 50 Hz for data acquisition. As DPF value is sensitive to age and wavelength, the wavelengths of near-infrared light used to collect the data collected were fixed in this study. In particular, we calculated the DPF value of each child according to the formula (DPF = 4.99 + 0.067^∗^Age^0.814^), which is more conducive to the data’s accuracy. After screening the data, the trials contained deformity or noisy data were treated as the incorrect trials and were discarded in advance of the formal analysis. The raw optical intensity data series were converted into changes in optical density (OD). The discrete wavelet transform was applied to every channel data series to remove motion artifacts, with the tuning parameter (α) of wavelet filtering set at 0.1. To reduce slow drifts and high-frequency noise, a bandpass filter (third-order Butterworth filter) with cut-off frequencies of 0.01–0.3 Hz (Delpy et al., 1988) was then applied to the data. The OD data were then converted into concentration changes of HbO and HbR. Among the three NIRS parameters measured, the concentration of HbO was found to be the most sensitive to changes in regional cerebral blood flow, which provided the strongest correlation with the blood oxygen level-dependent signal (Moriguchi and Hiraki, 2009). Thus, a change in the HbO concentration was considered to be the best indicator of brain activity. In the following analysis, only HbO concentration was calculated. Based on the previous research (Moriguchi and Hiraki, 2009), HbO concentration was converted into *z*-scores. The *z*-score was calculated using the mean value, and the *SD* of the HbO concentration changes during the rest phase.

Individual data were processed using MATLAB 2013b (Mathworks, MA, United States) (Huppert et al., 2009) and analyzed using *the Homer2 NIRS* processing package. The mean of z-scores (HbO) was calculated for each control task block and each MR task block separately for each participant. Then, the mean of z-scores (HbO) was calculated by averaging across the three task blocks for each participant. Finally, the mean of z-scores (HbO) across all channels were compared using *t*-tests between the High and Low groups using SPSS. The General Linear Model (GLM) analysis used to predict z-scores (HbO) in each channel was conducted in *R* (*Y*_Δ*HbO*_ = *aX*_*time*_ + *b*).

## Results

### Behavioral Results

All 38 participants completed the *Working Memory Assessment* (WMC scored between 3 and 10, *M*_*age*_ = 5.0, *SD* = 1.95). They were divided into high-WMC and low-WMC groups based on their WMC scores. Altogether nine participants were scored at least 0.5 *SD* higher than the mean, thus were included in the High-WMC group, whereas 18 were scored at 0.5 *SD* lower than the mean were included in the Low-WMC group, and the other 11 children were around the mean level, thus were excluded from this study. The MR task score of the High-WMC group (*M_*mr*_* = 23.44, *SD* = 0.882) was slightly lower than that of the Low-WMC group (*M*_*mr*_ = 23.67, *SD* = 0.594), *p* = 0.084. No significant age difference was found between the High-WMC and Low-WMC groups, *t* = 0.29, *p* = 0.774. Next, Spearman correlation analysis was conducted between the WMC and MR scores. A significant negative correlation was found in the Low-WMC group (*r* = −0.55, *p* < 0.05). In contrast, no significant correlation was found in the High-WMC group and Total samples, as shown in Table 1. This result indicated that generally, the MR task’s performance was not significantly correlated with their working memory capacity.

**TABLE 1:** Descriptive and correlational statistics of the low-MWC (*N*_1_ = 18) and high-WMC (*N*_2_ = 9) groups.

	Working memory	Mental rotation	WM/MR correlation	Sig (2-tailed)
	Mean (SD)	Mean (SD)		
High-WMC	7.89 (1.69)	23.44 (0.88)	0.46	0.212
Low-WMC	3.56 (0.51)	23.67 (0.59)	−0.55*	0.018*
Total	5.0 (1.95)	23.29 (1.41)	−0.23	0.168

*p < 0.05; ** p < 0.01; *** p < 0.001.

### *T*-Tests Results

First, a set of two-sample (independent groups) *t*-tests was conducted to determine any significant difference in the mean HbO increase between the High and Low-WMC groups in the control task. As multiple channels were involved in this type of *t*-tests, all the results were corrected for multiple comparisons using the false discovery rate (FDR), and the adjusted significant level of *p*-value was set at 0.05. The results indicated a significant between-group difference in BA 9 (ch 12) (*t* = 3.085, *p* < 0.01), BA 10 (ch 13) (*t* = 2.416, *p* < 0.05), BA 10 (ch 15) (*t* = 3.079, *p* < 0.01). As shown in Table 2, a significant increase in HbO was observed in BA 9 and BA 10 in the Low-WMC group compared to the High-WMC group.

**TABLE 2:** Comparison of increases in HbO between the low-MWC (*N*_1_ = 18) and high-WMC (*N*_2_ = 9) groups in the control task.

	Group	Mean	*SD*	*T*-value	*p*-value
Channel 1	Low	–0.463	1.381	1.681	0.105
	High	–1.472	1.644		
Channel 2	Low	–0.559	2.067	–1.124	0.272
	High	0.279	1.148		
Channel 3	Low	–1.248	1.725	0.359	0.722
	High	–1.530	2.283		
Channel 4	Low	–0.909	1.610	–0.291	0.774
	High	–0.719	1.570		
Channel 5	Low	–0.542	2.067	0.181	0.858
	High	–0.698	2.203		
Channel 6	Low	–0.078	1.055	–0.970	0.341
	High	0.306	0.762		
Channel 7	Low	–0.982	1.233	–0.357	0.724
	High	–0.794	1.413		
Channel 8	Low	–1.285	2.182	–0.863	0.396
	High	–0.514	2.201		
Channel 9	Low	–0.024	0.608	–0.551	0.586
	High	0.099	0.379		
Channel 10	Low	1.412	1.928	1.605	0.121
	High	0.241	1.445		
Channel 11	Low	0.220	1.604	1.684	0.105
	High	–0.900	1.685		
Channel 12	Low	0.122	1.234	3.085	0.005**
	High	–1.553	1.514		
Channel 13	Low	0.188	1.325	2.416	0.023*
	High	–1.138	1.382		
Channel 14	Low	–0.665	2.195	–0.287	0.776
	High	–0.427	1.639		
Channel 15	Low	0.214	1.375	3.079	0.005**
	High	–1.880	2.158		
Channel 16	Low	0.148	1.130	1.136	0.267
	High	–0.622	2.428		
Channel 17	Low	–0.331	1.260	0.672	0.508
	High	–0.669	1.177		

*p < 0.05; **p < 0.01; ***p < 0.001.

Next, a set of two-sample (independent groups) *t*-tests was conducted to determine any significant difference in the mean HbO increase between the High and Low-WMC groups in the MR task. The results indicated a significant between-group difference in BA 44 (ch 7) (*t* = −2.349, *p* < 0.05), BA 44 (ch 8) (*t* = −2.206, *p* < 0.05), BA 9 (ch 14) (*t* = −2.261, *p* < 0.05), BA 9 (ch 16) (*t* = 2.149, *p* < 0.05). As shown in Table 3, the High-WMC group had significantly more increase in HbO than the Low-WMC group in BA 44 (ch 7 and 8) and BA 9 (ch 14), indicating more brain activation in these areas. However, a significantly less increase in BA 9 (ch 16) was observed in the High-WMC than the Low-WMC group.

**TABLE 3:** Comparison of increases in HbO between the low-MWC (*N*_1_ = 18) and high-WMC (*N*_2_ = 9) groups in the MR task.

	Group	Mean	SD	*T*-value	*p*-value
Channel 1	Low	–1.185	2.433	–0.790	0.437
	High	–0.292	3.380		
Channel 2	Low	–1.319	2.328	–1.907	0.068
	High	0.773	3.324		
Channel 3	Low	–2.077	1.858	–1.022	0.317
	High	–1.215	2.451		
Channel 4	Low	–1.524	2.296	–1.059	0.300
	High	–0.610	1.666		
Channel 5	Low	–1.328	2.025	–1.224	0.232
	High	–0.094	3.215		
Channel 6	Low	0.593	1.565	0.707	0.486
	High	0.170	1.220		
Channel 7	Low	–1.943	2.342	–2.349	0.028*
	High	–0.202	1.486		
Channel 8	Low	–2.502	2.233	–2.206	0.037*
	High	–0.538	2.064		
Channel 9	Low	–0.118	0.689	–0.128	0.899
	High	–0.087	0.304		
Channel 10	Low	1.665	2.869	0.110	0.914
	High	1.534	3.032		
Channel 11	Low	–0.355	2.128	–0.202	0.842
	High	–0.157	2.893		
Channel 12	Low	0.461	1.266	2.027	0.053
	High	–0.900	2.244		
Channel 13	Low	–0.375	2.342	–0.643	0.526
	High	0.162	1.209		
Channel 14	Low	–1.136	2.585	–2.261	0.034*
	High	0.741	1.691		
Channel 15	Low	–0.016	2.256	0.720	0.478
	High	–0.612	1.438		
Channel 16	Low	0.353	2.399	2.149	0.044*
	High	–1.452	1.865		
Channel 17	Low	0.132	1.606	0.157	0.876
	High	0.006	2.573		

*p < 0.05; **p < 0.01; ***p < 0.001.

Third, a set of paired-samples *t*-tests was conducted to determine whether there were significant differences in the mean HbO increase between the MR and control tasks in the Low-WMC group. As shown in Table 4, no significant differences were found between the control and MR tasks in all the channels, *t*s > −1.834, *p*s > 0.084.

**TABLE 4:** Comparison of increases in HbO of the low-WMC group between the control and MR task.

MR—control	Paired differences		*T*-value	*p*-value
	Mean	Std. deviation		
Channel 1	–0.722	2.474	–1.239	0.232
Channel 2	–0.760	2.508	–1.286	0.216
Channel 3	–0.829	1.918	–1.834	0.084
Channel 4	–0.616	2.315	–1.128	0.275
Channel 5	–0.785	2.162	–1.542	0.142
Channel 6	0.671	1.627	1.749	0.098
Channel 7	–0.961	2.730	–1.494	0.154
Channel 8	–1.217	3.427	–1.507	0.150
Channel 9	–0.095	0.788	–0.510	0.617
Channel 10	0.253	2.970	0.361	0.722
Channel 11	–0.575	2.633	–0.926	0.367
Channel 12	0.338	1.939	0.740	0.469
Channel 13	–0.563	2.600	–0.919	0.371
Channel 14	–0.470	2.700	–0.739	0.470
Channel 15	–0.229	2.654	–0.366	0.719
Channel 16	0.206	1.744	0.500	0.623
Channel 17	0.463	2.152	0.913	0.374

*p < 0.05; **p < 0.01; ***p < 0.001.

Fourth, a set of paired-samples *t*-tests was conducted to determine whether there were significant differences in the mean HbO increase between the MR and control tasks in the High-WMC group. As shown in Table 5, a significant increase in HbO was found in BA 10 (ch 13) when comparing the MR task against the control one, *t* = 2.584, *p* < 0.05.

**TABLE 5:** Comparison of increases in HbO of the high-WMC group between the control and MR task.

MR—control	Paired differences		*T*-value	*p*-value
	Mean	Std. deviation		
Channel 1	1.180	2.674	1.324	0.222
Channel 2	0.494	2.604	0.569	0.585
Channel 3	0.315	0.884	1.068	0.316
Channel 4	0.109	1.525	0.215	0.835
Channel 5	0.604	2.596	0.698	0.505
Channel 6	–0.136	0.768	–0.531	0.610
Channel 7	0.592	0.986	1.803	0.109
Channel 8	–0.024	2.322	–0.031	0.976
Channel 9	–0.186	0.476	–1.175	0.274
Channel 10	1.293	2.696	1.439	0.188
Channel 11	0.743	3.132	0.712	0.497
Channel 12	0.653	2.237	0.875	0.407
Channel 13	1.301	1.510	2.584	0.032*
Channel 14	1.167	2.366	1.480	0.177
Channel 15	1.268	2.473	1.538	0.163
Channel 16	–0.830	2.527	–0.986	0.353
Channel 17	0.675	2.017	1.005	0.344

*p < 0.05; **p < 0.01; ***p < 0.001.

### Modeling HbO Increase for High and Low-MWC Groups in MR and Control Tasks

**FIGURE 5:**
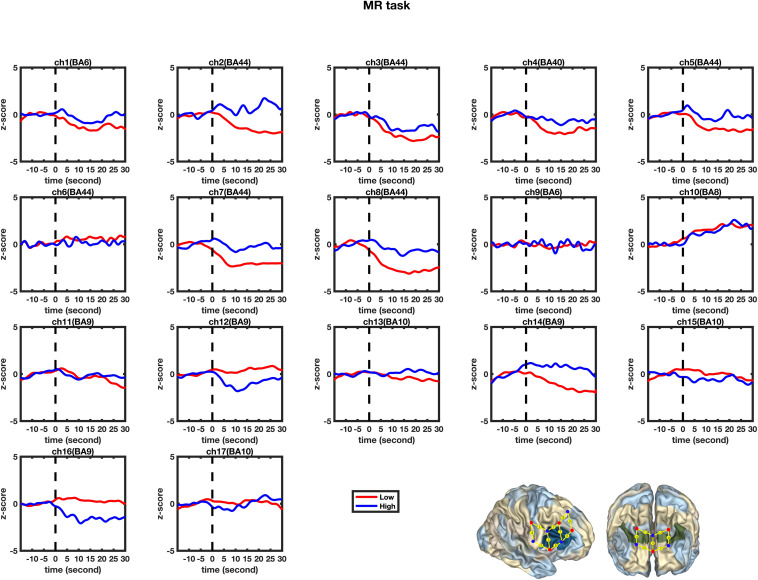
Temporal changes in the HbO concentration in the 17 channels during the mental rotation tasks. From left to right are channels 1–17. The HbO data for Low-MWC (*N*_1_ = 18) and High-WMC (*N*_2_ = 9) are shown in the blue and red lines, respectively.

**FIGURE 6:**
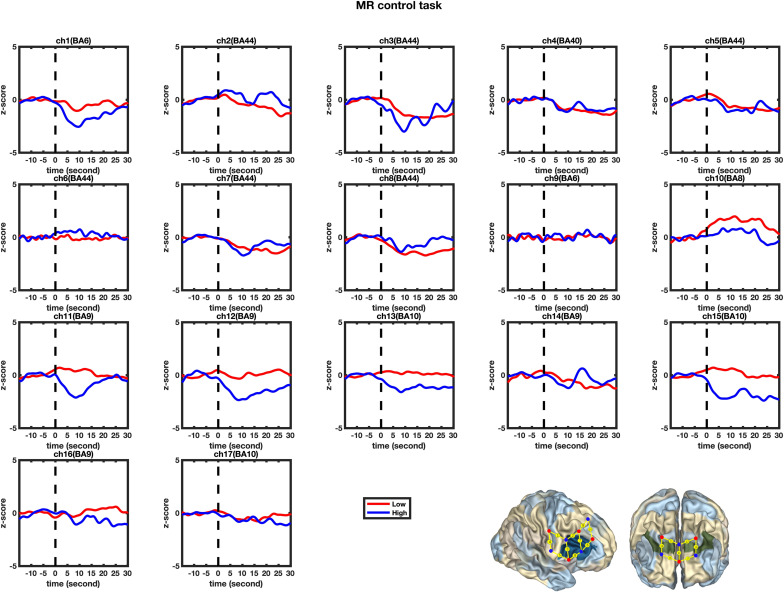
Temporal changes in the HbO concentration in the 17 channels during the Control tasks. From left to right are channels 1–17. The HbO data for the Low-MWC (*N*_1_ = 18) and High-WMC (*N*_2_ = 9) are shown in the blue and red lines, respectively.

A set of GLM analyses was conducted to model the change in HbO in the 17 channels based on *experiment time* for the High and Low = WMC groups, respectively. First, the changes in HbO during the MR and control tasks were analyzed for the Low group. As shown in Table 6 and Figure 5, during the MR task, significant HbO increase was observed in BA 6 (ch 9) [β = 0.41, Δ*R*^2^ = 0.26, *F* = 29.16 (for the model), *t* = 5.40 (for β), *p*s < 0.001], BA 8 (ch 10)[β = 0.84, Δ*R*^2^ = 0.70, *F* = 353.92 (for the model), *t* = 18.81 (for β), *p*s < 0.001], BA 9 (ch 12) [β = 0.69, Δ*R*^2^ = 0.47, *F* = 133.77 (for the model), *t* = 11.57 (for β), *p*s < 0.001], and BA 44 (ch 6) [β = 0.40, Δ*R*^2^ = 0.16, *F* = 28.39 (for the model), *t* = 5.33 (for β), *p*s < 0.001]. Meanwhile, significant decreases were found in the other channels including BA 6 (ch 1), BA 40 (ch 4), BA 44 (ch 2, 3, 5, 7, and 8), BA 9 (ch 11, 14, and16), BA 10 (ch 13, 15, and 17), *F*s > 16.45 (for the models), *t*s < −4.06 (for β), *p*s < 0.001. All these results jointly indicated that **BA6, BA8, BA9, and BA 44** were significantly activated during the MR task in this Low-WMC group. During the control task, as shown in Table 7 and Figure 6, significant HbO increase was observed in BA 9 (ch 12) [β = 0.51, Δ*R*^2^ = 0.26, *F* = 51.88 (for the model), *t* = 7.21 (for β), *p*s < 0.001], and BA 9 (ch 16) [β = 0.73, Δ*R*^2^ = 0.53, *F* = 167.78 (for the model), *t* = 12.95 (for β), *p*s < 0.001]. Meanwhile, significant decrease in HbO was observed in BA 44 (ch 2, 3, 5, 7, and 8), BA 40 (ch 4), BA 8 (ch 10), BA 9 (ch 11 and 14), BA 10 (ch 13 and 15), *F*s > 39.35 (for the models), *t*s < −6.27 (for β), *p*s < 0.01. BA 9 was significantly activated during the control task in the Low-WMC group. After controlling for the activation of BA 9 in the control tasks, we could conclude that **BA6, BA8, and BA 44 were significantly activated during the MR task in this Low-WMC group.**

**TABLE 6:** Predicting increase in HbO for the low group (*N*_1_ = 18) in the MR task.

	β	Δ*R*^2^	*F*-value	*T-*value
Channel 1	–0.590	0.343	78.865***	−8.881***
Channel 2	–0.915	0.836	762.619***	−27.616***
Channel 3	–0.794	0.628	252.429***	−15.888***
Channel 4	–0.491	0.236	47.090***	−6.862***
Channel 5	–0.788	0.618	242.561***	−15.574***
Channel 6	0.401	0.155	28.394***	5.329***
Channel 7	–0.506	0.251	51.031***	−7.144***
Channel 8	–0.687	0.468	132.096***	−11.493***
Channel 9	0.406	0.159	29.158***	5.400***
Channel 10	0.840	0.703	353.924***	18.813***
Channel 11	–0.927	0.858	898.024***	−29.967***
Channel 12	0.689	0.471	133.768***	11.566***
Channel 13	–0.900	0.809	630.593***	−25.111***
Channel 14	–0.968	0.936	2193.944***	−46.840***
Channel 15	–0.928	0.860	912.931***	−30.215***
Channel 16	–0.833	0.692	335.364***	−18.313***
Channel 17	–0.316	0.094	16.455***	−4.056***

*p < 0.05; **p < 0.01; *** p < 0.001.

**TABLE 7:** Predicting increase in HbO for the low-WMC Group (*N*_1_ = 18) in the control task.

	β	Δ*R*^2^	*F*-value	*T-*value
Channel 1	0.063	0.004	0.594	0.770
Channel 2	–0.961	0.923	1784.655	−42.245***
Channel 3	–0.760	0.574	201.923	−14.210***
Channel 4	–0.898	0.806	618.974***	−24.879***
Channel 5	–0.876	0.766	489.510***	−22.125***
Channel 6	0.031	–0.006	0.145	0.381
Channel 7	–0.833	0.691	334.929***	−18.301***
Channel 8	–0.476	0.221	43.299***	−6.580***
Channel 9	0.075	–0.001	0.844	0.919
Channel 10	–0.458	0.205	39.346***	−0.6.273***
Channel 11	–0.931	0.865	959.229***	−30.971***
Channel 12	0.509	0.255	51.877***	7.209***
Channel 13	–0.809	0.653	281.010***	−16.763***
Channel 14	–0.897	0.804	610.320***	−24.705***
Channel 15	–0.889	0.788	556.363***	−23.587***
Channel 16	0.729	0.528	167.783***	12.953***
Channel 17	–0.013	–0.007	0.026	–0.161

*p < 0.05; **p < 0.01;***p < 0.001.

Second, the changes in HbO during the control and MR tasks were analyzed for the High-WMC group. During the MR task, as shown in Table 8 and Figure 5, significant increase in HbO were observed in BA 8 (ch 10) [β = 0.87, Δ*R*^2^ = 0.75, *F* = 456.59 (for the model), *t* = 21.37 (for β), *p*s < 0.001], BA 10 (ch 13) [β = 0.35, Δ*R*^2^ = 0.12, *F* = 21.17 (for the model), *t* = 4.60 (for β), *p*s < 0.001], BA 10 (ch 17) [β = 0.86, Δ*R*^2^ = 0.74, *F* = 416.19 (for the model), *t* = 20.40 (for β), *p*s < 0.001], and BA 44 (ch 2) [β = 0.336, Δ*R*^2^ = 0.12, *F* = 21.90 (for the model), *t* = 4.68 (for β), *p*s < 0.001]. Meanwhile, significant HbO decrease was observed in BA 6 (ch 9), BA 9 (ch 11, 14, and16), BA 10 (ch 15), BA 40 (ch 4), and BA 44 (ch 3, 5, 7, and 8), *F*s > 14.69 (for the models), *t*s < −3.83 (for β), *p*s < 0.001. All these results jointly indicated that **BA8, BA10, and BA 44** were significantly activated during the MR task in this High-WMC group. During the control task, as shown in Table 9 and Figure 6, significant HbO increase was observed in BA 6 (ch 1) [β = 0.32, Δ*R*^2^ = 0.09, *F* = 16.35 (for the model), *t* = 4.04 (for β), *p*s < 0.001], BA 9 (ch 11) [β = 0.55, Δ*R*^2^ = 0.30, *F* = 65.61 (for the model), *t* = 8.10 (for β), *p*s < 0.001], BA 44 (ch 3) [β = 0.32, Δ*R*^2^ = 0.10, *F* = 16.71 (for the model), *t* = 4.09 (for β), *p*s < 0.001], BA 44 (ch 8) [β = 0.37, Δ*R*^2^ = 0.13, *F* = 23.92 (for the model), *t* = 4.89 (for β), *p*s < 0.001]. Meanwhile, significant decrease in HbO was observed in BA 8 (ch 10), BA 9 (ch 16), BA 10 (ch 13, 15, and 17), BA 40 (ch 4), and BA 44 (ch 2, 5, and 6), *F*s > 11.14 (for the models), *t*s < −3.34 (for β), *p*s < 0.01. BA6, BA9, and BA 44 were significantly activated during the control task in the High-WMC group. After controlling for the activation of BA6, 9, 44 in control tasks, we could conclude that BA8 and BA10 were significantly activated during the MR task in this High-WMC group.

**TABLE 8:** Predicting increase in HbO for the high-WMC group (*N*_2_ = 9) in the MR task.

	β	Δ*R*^2^	*F*-value	*T-*value
Channel 1	–0.147	0.015	3.263	–1.806
Channel 2	0.359	0.123	21.903***	4.680***
Channel 3	–0.591	0.345	79.493***	−8.916***
Channel 4	–0.496	0.241	48.184***	−6.941***
Channel 5	–0.426	0.176	32.874***	−5.734***
Channel 6	0.021	–0.006	0.064	0.253
Channel 7	–0.330	0.103	18.111***	−4.256***
Channel 8	–0.630	0.393	97.391***	−9.869***
Channel 9	–0.400	0.154	28.175***	−5.308***
Channel 10	0.869	0.754	456.594***	21.368***
Channel 11	–0.396	0.157	27.461***	−5.240***
Channel 12	0.100	0.003	1.491	1.221
Channel 13	0.354	0.119	21.174***	4.601***
Channel 14	–0.806	0.646	273.464***	−16.537***
Channel 15	–0.301	0.084	14.698***	−3.834***
Channel 16	–0.574	0.325	72.593***	−8.520***
Channel 17	0.859	0.736	416.189***	20.401***

*p < 0.05; **p < 0.01; ***p < 0.001.

**TABLE 9:** Predicting increase in HbO for the high-WMC group (*N*_2_ = 9) in the MR control task.

	β	Δ*R*^2^	*F*-value	*T-*value
Channel 1	0.315	0.093	16.350***	4.043***
Channel 2	–0.612	0.370	88.648***	−9.415***
Channel 3	0.318	0.095	16.708***	4.088***
Channel 4	–0.569	0.320	71.006***	−8.427***
Channel 5	–0.405	0.158	28.989***	−5.384***
Channel 6	–0.799	0.636	261.558***	−16.173***
Channel 7	0.119	0.008	2.131	1.460
Channel 8	0.373	0.133	23.923***	4.891***
Channel 9	–0.128	0.010	2.467	–1.571
Channel 10	–0.633	0.396	98.696***	−9.935***
Channel 11	0.554	0.302	65.605***	8.100***
Channel 12	0.145	0.015	3.200	1.789
Channel 13	–0.265	0.064	11.143**	−3.338**
Channel 14	–0.050	–0.004	0.376	–0.613
Channel 15	–0.482	0.227	44.842***	−6.696***
Channel 16	–0.743	0.549	182.532***	−13.510***
Channel 17	–0.823	0.675	309.806***	−17.601***

*p < 0.05; **p < 0.01; ***p < 0.00.

## Discussion

### Neural Correlates of Mental Rotation in the Low-WMC Group

First, this study found no significant differences in the activation of the 17 channels between the Low group’s control and MR tasks. This result indicated that the Low-WMC group activated the same brain areas to complete both the control and MR tasks. As the MR task required the successful processing of mental rotation, whereas the control task did not, this result indicated that mental rotation’s neural correlate might be involved in completing both MR and control tasks in this Low group. In addition, when they performed the MR task, they demonstrated significant deactivation in BA 40 and BA 44, indicating that both BA 40 and BA 44 might play a critical role in completing the mental rotation task. This finding is consistent with that of Wu et al. (2020).

Second, the GLM analysis found that BA6, BA8, BA9, and BA 44 were significantly activated during the MR task in the Low-WMC group, whereas only BA9 was significantly activated during the control task. After controlling for the effect of BA9 in the control task, we could conclude that BA6, BA8, and BA 44 were significantly activated during the MR task in the Low-WMC children.

All these findings jointly indicated that: (1) BA 9, as one of the neural cores of executive function, was consistently involved in the processing of both MR and control tasks; (2) BA 44 was substantially involved in mental rotation (but not in the control task) in Low-WMC children, demonstrating that BA 44 could be one of the core neural correlates for the manipulation of mental rotation; and (3) BA6, BA8, and BA 40 might also be involved in the mental rotation for the Low-WMC group.

### Neural Correlate of Mental Rotation in the High-WMC Group

First, this study found a significant HbO increase in BA10 (when comparing the MR against the control task) in the High-WMC group. This result indicated that the High-WMC group had to activate BA10 more significantly to complete the mental rotation task than their brain activation during the control task. Second, the GLM results indicated that BA8, BA10, and BA 44 were significantly activated during the MR task, whereas BA6, BA9, and BA44 were significantly activated during the control tasks. Therefore, we can conclude that: (1) BA 44 has been substantially involved in the processing of both the MR and control tasks, demonstrating that BA 44 is one of the core neural correlates for the manipulation of mental rotation; (2) BA8 and BA10 were significantly activated during the MR task in the High-WMC group; (3) BA6 and BA9 were responsible for the manipulation of the control task, which requires visual-spatial information processing, movement planning, hand movement (Krüger et al., 2014).

### The Roles of BA9, BA10, and BA 10 in Working Memory and Mental Rotation

This study found two different patterns of neural correlates of MR for the Low-WMC and High-WMC preschoolers, respectively. The Low-WMC group tended to activate BA6, BA8, and BA44 when processing the MR tasks, whereas the High-WMC activated BA8, BA10, and BA 44. The comparison of Low-WMC and High-WMC patterns indicated the significant differences in the activation of BA9 and BA44. This finding implies that BA9 and BA44 might play essential roles in the collaboration between mental rotation and working memory.

First, BA 44 functions significantly in binding the language elements, selecting information among competing sources, generating/extracting action meanings, and cognitive control mechanisms for the syntactic processing of sentences (Aron et al., 2004). Also, BA 44 is responsible for both hand movements (Rizzolatti et al., 2002) and cognitive shifting in the Dimensional Change Card Sort (DCCS) task in preschool children (Moriguchi and Hiraki, 2009; Wu et al., 2020). In this study, hand movement is critical to successfully completing all these MR tasks, as the children should make appropriate hand movements to return the stimuli to the correct place. Therefore, we added a control experiment and found that BA44 was consistently involved in MR and control tasks in the High-WMC group (but not in the Low group). This nuanced difference indicated that BA44 should be responsible for the mental rotation rather than hand movement in this study. All the participants were right-handed, and we only tested the right hemisphere BA44. This finding has provided empirical support to that of Wu et al. (2020) that BA44 should be regarded as one of the core neural correlates of mental rotation in preschoolers.

Also, in the High-WMC group, BA 10 was a significant activated area for the mental rotation. BA10 is extensively involved in cognitive processing in the human brain, but its function is poorly understood. A meta-analysis found that it involved working memory, episodic memory, and multiple-task coordination (Gilbert et al., 2006). Therefore, this finding indicates that working memory is substantially involved in MR tasks; thus, BA 10 plays a significant role in mental rotation. This is consistent with that of Hyun and Luck (2007), who found that prefrontal areas (BA 9 and BA 10) were responsible for the control and manipulation of information in working memory and provides partial evidence to support our hypothesis that BA 9 and BA 10 might play a critical role in the processing of mental rotation tasks.

Last, this study found that the High and Low-WMC groups differed significantly in BA 9 during mental rotation. BA 9 is widely involved in attributing intention, theory of mind, working memory, spatial memory, recognition, recall, and planning (Brunet et al., 2000; Gallagher et al., 2002; Leung et al., 2002; Pochon et al., 2002; Raye et al., 2002; Zhang et al., 2003). In the mental rotation, the High-WMC group had significantly more activation in BA9 than the Low-WMC group, indicating that BA9 might be relevant to the between-group differences in working memory capacity. This finding implies that BA9 might also play an essential role in preschoolers’ working memory and mental rotation, which is also consistent with that of Wu et al. (2020).

## Conclusion, Limitations, and Implications

In summary, this study found two patterns of neural correlates of mental rotation for the preschoolers with low and high working memory capacity. The Low-WMC group tended to activate BA6, BA8, and BA44, whereas the High-WMC group activated BA8, BA10, and BA 44 when processing the MR tasks. The significant differences in the activation of BA9 and BA44 between the Low and High-WMC patterns indicated the two areas might play essential roles in the collaboration between mental rotation and working memory. In addition, the High-WMC group has demonstrated significant activation in BA 10, indicating that BA10 might be specifically responsible for the mental rotation when completing the MR tasks. In contrast, the Low-WMC group had no significant activation in any studied areas compared to the control task. All these findings jointly indicate that BA9 and BA10 might play a vital role in processing both working memory and mental rotation, and BA44 might be one of the core neural correlates of mental rotation in preschoolers (Wu et al., 2020).

This study has some limitations. First, other brain regions, especially left frontal areas, might also contribute to mental rotation development. However, with minimal channels, this study could only focus on the right IFC and inferior prefrontal areas. Second, the younger (3-year-olds) and older (7-year-olds) should have been included in this study to examine whether the neural network of mental rotation could be matured and adultlike in primary school years. Last, this study found a significant negative correlation between WMC and MR scores in the Low-WMC group. Is this finding caused by sample bias or developmental differences? This cross-sectional study with a small sample was incapable of address this question. Further studies with more samples of varying ages should be conducted in the future. Therefore, we need to address all these limitations by investigating brain activation using various tasks and longitudinal designs in the future.

Nevertheless, the findings of this study do have some implications for future studies. First, preschoolers can complete the mental rotation tasks, and future studies can further explore the neural correlates of mental rotation using this research paradigm. Second, BA44 has been substantially involved in processing both the MR and control tasks in the High-WMC group, indicating that the role of BA 44 might be more complicated and deserves further exploration. Third, the critical roles of BA 9 and BA 10 in the processing of working memory and mental rotation should be further explored and verified with more experiments. Therefore, more empirical evidence could be provided to confirm the neural correlates of working memory in preschoolers.

## Data Availability Statement

The raw data supporting the conclusions of this article will be made available by the authors, without undue reservation.

## Ethics Statement

The studies involving human participants were reviewed and approved by the ethics committee of the Faculty of Medicine, Shenzhen University, China. Written informed consent to participate in this study was provided by the participants’ legal guardian/next of kin.

## Author Contributions

JY, DW, JL, and SX collected the data. DW and HL designed the experiment. JY and CC analyzed the data. JY, HL, DW, and CC drafted the manuscript. All authors contributed to the article and approved the submitted version.

## Conflict of Interest

The authors declare that the research was conducted in the absence of any commercial or financial relationships that could be construed as a potential conflict of interest.
